# The distribution of the genus *Sphecodes* Latreille (Hymenoptera, Halictidae) of the Arabian Peninsula and surrounding countries with description of hitherto unknown female of *S. atlanticus* Warncke, 1992 and male of *S. dathei* Schwarz, 2010

**DOI:** 10.3897/zookeys.872.35361

**Published:** 2019-08-20

**Authors:** Yulia V. Astafurova, Maxim Yu. Proshchalykin, Maximilian Schwarz

**Affiliations:** 1 Zoological Institute, Russian Academy of Sciences, Universitetskaya Nab., 1, Saint Petersburg 199034, Russia Zoological Institute, Russian Academy of Sciences Saint Petersburg Russia; 2 Federal Scientific Centre for East Asian Terrestrial Biodiversity, Far Eastern Branch of Russian Academy of Sciences, Vladivostok 690022, Russia Federal Scientific Centre for East Asian Terrestrial Biodiversity, Far Eastern Branch of Russian Academy of Sciences Vladivostok Russia; 3 A-4052, Ansfelden, Austria Unaffiliated Ansfelden Austria

**Keywords:** Anthophila, Apiformes, cleptoparasites, fauna, lectotype, taxonomy

## Abstract

This study summarises all available information on the bees of the genus *Sphecodes* in the Arabian Peninsula and surrounding countries (Israel, Jordan, and Syria). Twenty-six species are currently known from this area, while five species are newly recorded from the Arabian Peninsula: *Sphecodes atlanticus* Warncke, 1992 (Saudi Arabia, Yemen), *S. intermedius* Blüthgen, 1923 (UAE), *S. nomioidis* Pesenko, 1979 (UAE, Oman), *S. puncticeps* Thomson, 1870 (Saudi Arabia), and *S. turanicus* Astafurova & Proshchalykin, 2017 (Saudi Arabia). In addition, twelve species are newly recorded from Jordan, six for Syria, and four for Israel. The female of *S. atlanticus* Warncke, 1992 and the male of *S. dathei* Schwarz, 2010 are here described for the first time and a lectotype is designated for *S. intermedius* Blüthgen, 1923.

## Introduction

**Figure 1.**
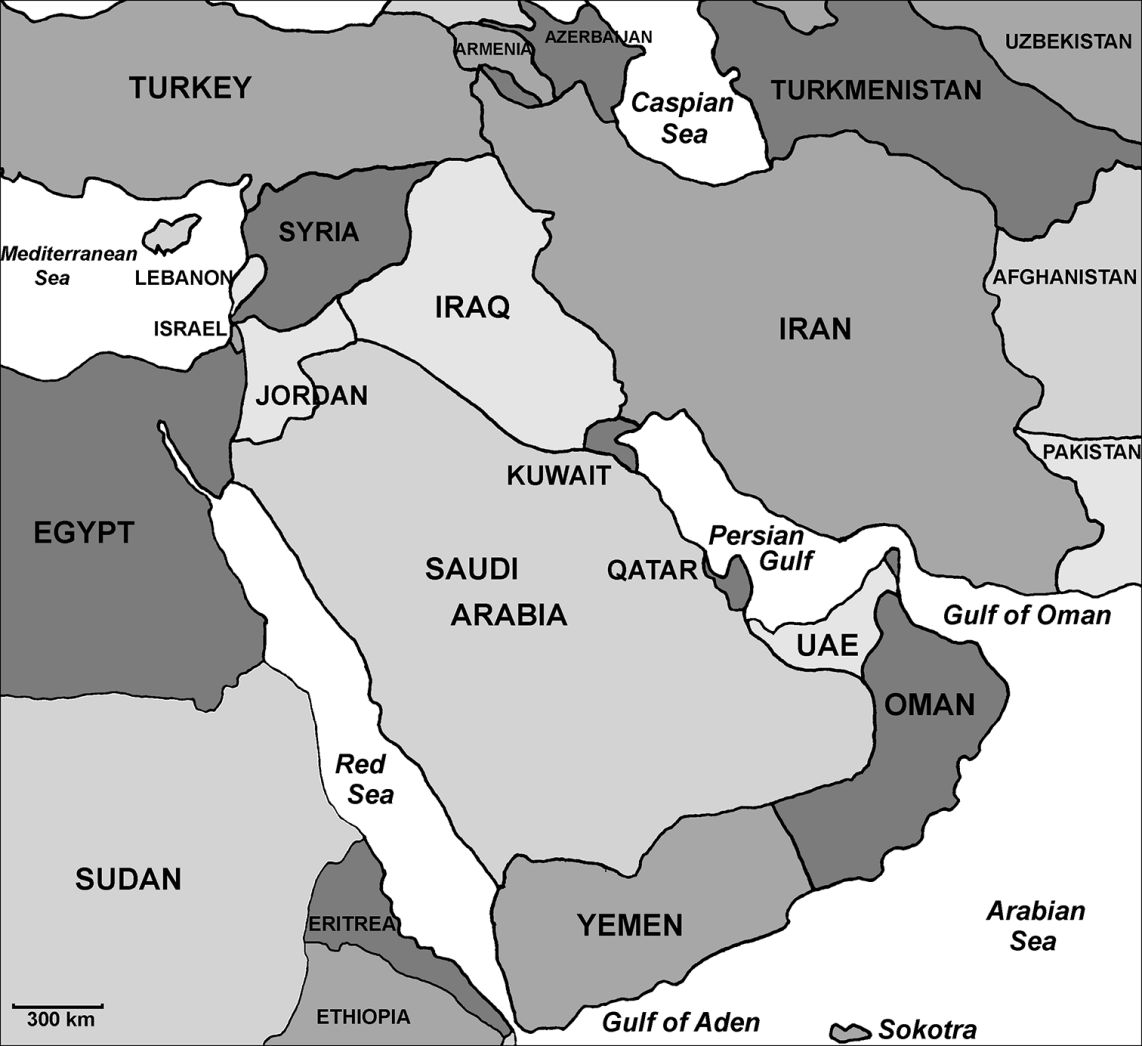
Map of the Arabian Peninsula and surrounding lands.

The present paper is part of a series of studies dealing with the bees of the genus *Sphecodes* of the territory of the Palaearctic region (Warncke 1992; Bogusch and Straka 2012; Özbek et al. 2015; Astafurova and Proshchalykin 2014, 2015a, b, c, 2016a, b, 2017a, b, c, 2018; Astafurova et al. 2014, 2015, 2018a, b, c, d). The goal of this survey is to improve the knowledge on the taxonomy and distribution of *Sphecodes* in the Arabian Peninsula and surrounding countries (Israel, Jordan and Syria) (Fig. 1) as an essential foundation for advanced biogeographical investigations.

For a long time, the Arabian bee fauna has been one of the lesser sampled faunas of the world. But in recent years significant progress has been made towards a better knowledge of the bees from the Arabian Peninsula, in particular regarding the family Halictidae (Dathe 2009, Engel et al. 2013). A first contemporary inventory of the Halictidae of the Arabian Peninsula was compiled by Ebmer (2008) and Dathe (2009). Later, additional species have been described and recorded by Pesenko and Pauly (2009), Schwarz (2010), Alqarni et al. (2014), Bossert (2017), and Ascher and Pickering (2019) so that there are currently 82 species from 13 genera of family Halictidae known from this area, but the *Sphecodes* fauna of Arabian Peninsula is particularly under-recorded.

Probably the first information on the genus *Sphecodes* Latreille from the Arabian Peninsula and its adjacent lands was published by Lepeletier de Saint Fargeau (Lepeletier de Saint Fargeau and Audinet-Serville 1825), who described *S. olivieri* from ‘Arabie’. Almost two centuries later, in his monograph on the Western Palaearctic *Sphecodes*, Warncke (1992) recorded several species from Israel, Syria and Lebanon (Table 1). The list of bees of the Arabian Peninsula published by Dathe (2009) included two *Sphecodes* species: *S. olivieri* and *S. longuloides* Blüthgen. In the recently published third volume of the “Arthropod fauna of UAE”, Schwarz (2010) described *S. dathei* and *S. villosulus* and recorded *S. marginatus* Hagens and *S. pinguiculus* Pérez from the United Arab Emirates. In total, nineteen *Sphecodes* species have been recorded from the Arabian Peninsula and its adjacent lands so far (Table 1). The genus *Sphecodes* is not yet documented from Kuwait, Bahrain, or Iraq. Clearly this cosmopolitan genus is present in these countries and it is only a matter of time before the fauna is sampled and recorded.

**Table 1.** Checklist of the *Sphecodes* species of the Arabian Peninsula and surrounding lands including distribution by countries.

Species		Arabian Peninsula					surrounding lands			
		UAE	Oman	Qatar	Saudi Arabia	Yemen	Lebanon	Israel	Jordan	Syria
1	*S. alternatus* Smith							○●	●	○
2	*S. atlanticus* Warncke				●	●				
3	*S. barbatus* Blüthgen									●
4	*S. dathei* Schwarz	○●			●	●				
5	*S. dusmeti* Blüthgen	○								
6	*S. ephippius* (Linnaeus)						○	○		
7	*S. gibbus* (Linnaeus)							○●	●	
8	*S. intermedius* Blüthgen	●						○●	●	
9	*S. longuloides* Blüthgen	○								
10	*S. longulus* Hagens							●	○●	○●
11	*S. majalis* Pérez								●	
12	*S. marginatus* Hagens	○						●	●	
13	*S. monilicornis* (Kirby)							○	○●	●
14	*S. nomioidis* Pesenko	●	●						○	
15	*S. olivieri* Lepeletier	○●	●	○	●			○●	●	
16	*S. pellucidus* Smith								●	●
17	*S. pinguiculus* Pérez	○	●		●			○●		●
18	*S. puncticeps* Thomson				●			○●	●	●
19	*S. rubicundus* Hagens							●		
20	*S. rubripes* Spinola							○	●	○
21	*S. ruficrus* (Erichson)							○	●	
22	*S. rufiventris* (Panzer)							○	●	
23	*S. tadschicus* Blüthgen							●		
24	*S. turanicus* Astafurova & Proshchalykin				●					
25	*S. schenckii* Hagens							○	●	●
26	*S. villosulus* Schwarz	○●			●					
**Total**:		9	4	1	7	2	1	16	15	9
		12					20			

White circle – published records (Meyer 1924; Warncke 1992; Dathe 2009; Schwarz 2010; Ascher and Pickering 2019); black circle – current data. Genus *Sphecodes* are not known in Kuwait, Bahrain, and Iraq.

Based on a comprehensive study of specimens in various collections, we here list 23 species of the genus *Sphecodes*, with five species recorded from the Arabian Peninsula for the first time. Additionally, twelve species are newly recorded from Jordan, six species newly recorded from Syria, and four species newly recorded from Israel. The female of *S. atlanticus* Warncke, 1992 and the male of *S. dathei* Schwarz, 2010 are here described for the first time and a lectotype is designated for *S. intermedius* Blüthgen, 1923.

## Materials and methods

The results presented in this paper are based on 235 specimens collected in the Arabian Peninsula and surrounding territories and currently housed in the Natural History Museum (London, UK, NHMUK); the Zoological Institute, Russian Academy of Sciences(St. Petersburg, Russia, ZISP); Museum für Naturkunde der Humboldt Universität zu Berlin, Germany (ZISP), Senckenberg Deutsches Entomologisches Institut, Müncheberg, Germany (SDEI), Oberösterreichisches Landesmuseum, Biologiezentrum, Linz, Austria (OLBL) and the private collection of Maximilian Schwarz (Ansfelden, Austria, OLBL/PCMS). The following acronyms are used for the collections where type specimens are deposited:

**BLCU**Utah State University, Bee Biology and Systematics Laboratory, Logan, Utah, USA;

**ISZP**Institute of Systematics and Evolution of Animals, Polish Academy of Sciences, Krakow, Poland;

**MNHN**Muséum National d’Histoire Naturelle Paris, France;

**MRSN**Museo Regionale di Scienze Naturali, Torino, Italy;

**MZLU**Lund University, Lund, Sweden;

**NHMUK** Natural History Museum, London, UK;

**ZMUK**University of Copenhagen, Zoological Museum, Copenhagen, Denmark;

**ZSN**Zoologische Staatssammlung, München, Germany.

The taxonomy and distribution of species follows that of Warncke (1992), Bogusch and Straka (2012), and Astafurova and Proshchalykin (2017b). Identification keys are available in Warncke (1992), Astafurova and Proshchalykin (2017b) or Astafurova et al. (2018b), except for the two recently described new species (*S. dathei* and *S. villosulus*). A detailed synonymy can be found in Astafurova and Proshchalykin (2016b, 2017b). Morphological terminology follows that of Engel (2001) and Michener (2007). The ventral surface of some flagellomeres bear a distinctive patch of sensilla trichodea A (sensu Årgent and Svensson 1982), which we refer to as ‘tyloids’, easily observable under the microscope. Abbreviations F, T, and S are used for flagellomere, metasomal tergum and metasomal sternum respectively. The density of integumental punctures is described using the following formula: puncture diameter (in μm) / ratio of distance between punctures to average puncture diameter, e.g., 15–20 μm / 0.5–1.5. Integumental sculpture other than distinctive surface punctation is described following Harris (1979): areolate – coarse, contiguous punctures; reticulate – superficially net-like or network of raised lines; rugose – irregular, nonparallel, wrinkled raised lines (rugae); rugulose – minutely rugose; strigate – narrow, transverse or longitudinal streaks (strigae), variety of parallel lineations; tessellate – regular network of shallow grooves with flat interspaces.

Specimens were studied with a Leica M205A stereomicroscope and photographs taken with a combination of stereomicroscope (Olympus SZX10) and digital camera (Canon EOS70D). Final images are stacked composites using the program Helicon Focus 6. All images were post-processed for contrast and brightness using Adobe Photoshop.

New distributional records are noted with an asterisk (*).

## Taxonomy

### List of species

#### Sphecodes alternatus

Taxon classificationAnimaliaHymenopteraHalictidae

Smith, 1853

C46A4EBF76A657D3964CC99B537BC064

Sphecodes alternatus Smith, 1853: 36, ♀ (syntypes: ♀♀, Albania; NHMUK).
Sphecodes punctiventris Hagens, 1882; S. gracilior Morawitz, 1893; S. antigae Tournier, 1901; S. reticulatus var. algeriensis Alfken, 1914; S. alternatus lindbergi Pittioni, 1950 (Synonyms).

##### Diagnosis.

See Astafurova et al. 2018a: 6.

##### Material examined.

ISRAEL: 1 ♀, Rehovot s.l., 29.IV.1975, K.M. Guichard (NHMUK 013380375); JORDAN: 1 ♀, 10 km N Petra, 3.V.1996, M. Halada (OLBL/OLBL/PCMS).

##### Published records.

Warncke 1992: 47, map (Israel, Syria); Ascher and Pickering 2019 (Israel).

##### Distribution.

Israel, *Jordan, Syria; North Africa, South and Central Europe, Russia (east to Khakassia Republic), Turkey, Caucasus, Iran, Central Asia, Kazakhstan, NW China.

#### Sphecodes atlanticus

Taxon classificationAnimaliaHymenopteraHalictidae

Warncke, 1992

F3062642680F57EA9FB353FA0E5078C1

Figures 5 , 9 , 10 , 19 , 22

Sphecodes atlanticus Warncke, 1992: 25, Abb. 17, ♂ (holotype: ♂, Algeria: Hoggar-Geb., Guelta; OLBL/PCMS), examined.

##### Diagnosis.

This species is similar to the Trans-Palaearctic *Sphecodes scabricollis* Wesmael, 1835 owing to the flat genal area, the developed preoccipital lateral carina, the densely punctate head and mesoscutum, the size and shape of male antennal tyloids, and in the similar gonostylar shape. However, *S. atlanticus* differs from *S. scabricollis* by a number of characters outlined in Table 2. In addition to presence of preoccipital lateral carina, *S. atlanticus* clearly differs from the *gibbus* species group (*S. anatolicus* Warncke, 1922, *S. gibbus* (Linnaeus, 1758), *S. nippon* Meyer, 1922, *S. rufiventris* (Panzer, 1798), *S. schenckii* Hagens, 1882, *S. tadschicus* Blüthgen in Popov, 1935; see Astafurova et al. 2018a) by a short distance from top of head to upper margin of lateral ocellus (2 lateral ocellar diameters as seen in dorsal view, versus those with a long vertex where this distance is at most 2.5–3.0 diameters).

**Table 2.** Differences between *Sphecodes atlanticus* Warncke, 1992 and *S. scabricollis* Wesmael, 1835.

Characters	*Sphecodes atlanticus*	*Sphecodes scabricollis*
**Both sexes**		
Distance from top of head to upper margin of lateral ocellus as seen in frontal and dorsal views	about one lateral ocellar diameters (Fig. 5)	about two lateral ocellar diameters (Fig. 3)
Propodeal triangle/metaposnotum	equal (in female) or longer (in male) than mesoscutellum (Figs 9, 10)	distinctly shorter than mesoscutellum (Figs 8)
Metasomal terga	with coarser and denser punctures (Fig. 19)	with fine and sparser punctures, especially on T1 (Fig. 18)
**Male**		
Mesoscutum	punctures separated by at most 1.5–2.0 puncture diameters; polished between punctures (Fig. 9)	areolate (Fig. 8)
Genitalia	gonocoxite dorsally with weak impression; gonostylar process longer (Fig. 22)	gonocoxite dorsally without impression; gonostylar process shorter (Fig. 23)
**Female**		
Paraocular areas	with dense pubescence obscuring integument (Fig. 5)	with sparse pubescence not obscuring integument (Fig. 3)

##### Description of hitherto unknown female.

Total body length 6.5–8.5 mm. Head (Fig. 5) black (except reddish mouthparts); transverse, 1.3 times as wide as long; vertex elevated, distance from top of head to upper margin of lateral ocellus ca. one lateral ocellar diameter as seen in frontal view and ca. two lateral ocellar diameters as seen in dorsal view; F1 and F2 transverse, 0.7–0.8 times as long as wide; F3 as long as wide; face with fine contiguous punctures (10–20 μm), clypeus with shiny interspaces between punctures separated by 0.1–0.5 of a puncture diameter; mandible with an inner tooth; paraocular areas and upper part of gena with dense adpressed, snow-white, plumose pubescence obscuring the integument.

**Figures 2–7.**
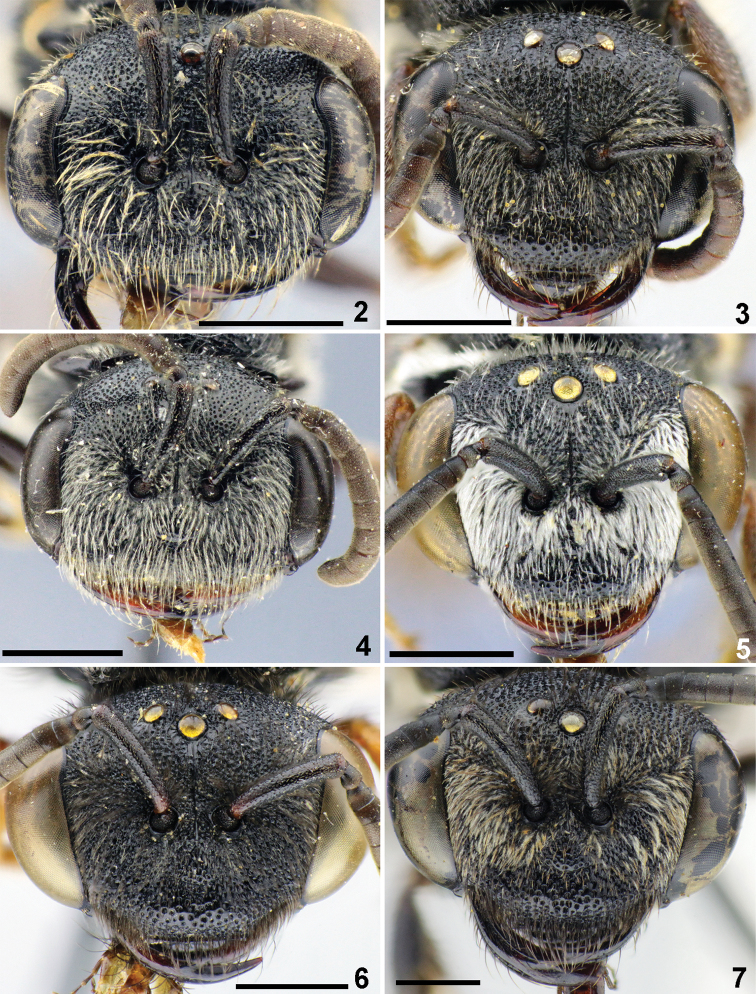
Head, females, frontal view. **2***Sphecodes majalis* Pérez **3***S. scabricollis* Wesmael **4***S. barbatus* Blüthgen **5***S. atlanticus* Warncke **6***S. rubripes* Spinola **7***S. albilabris* (Fabricius). Scale bars: 1.0 mm.

Mesosoma black; mesoscutum with coarse punctures (25–50 μm) separated by at most a puncture diameter (Fig. 10); mesoscutellum with irregular punctures separated by 0.1–4 puncture diameters; mesepisternum densely reticulate-rugose; propodeal triangle coarsely reticulate-rugose with large shiny, smooth interspaces between wrinkles (Fig. 10); lateral parts of propodeum finely and densely strigate or strigate-rugose with granulate interspaces between wrinkles; vertical part of propodeum smooth with coarse and dense punctures; legs reddish or dark brown. Hind wing costal margin with 9–10 hamuli.

**Figures 8–11.**
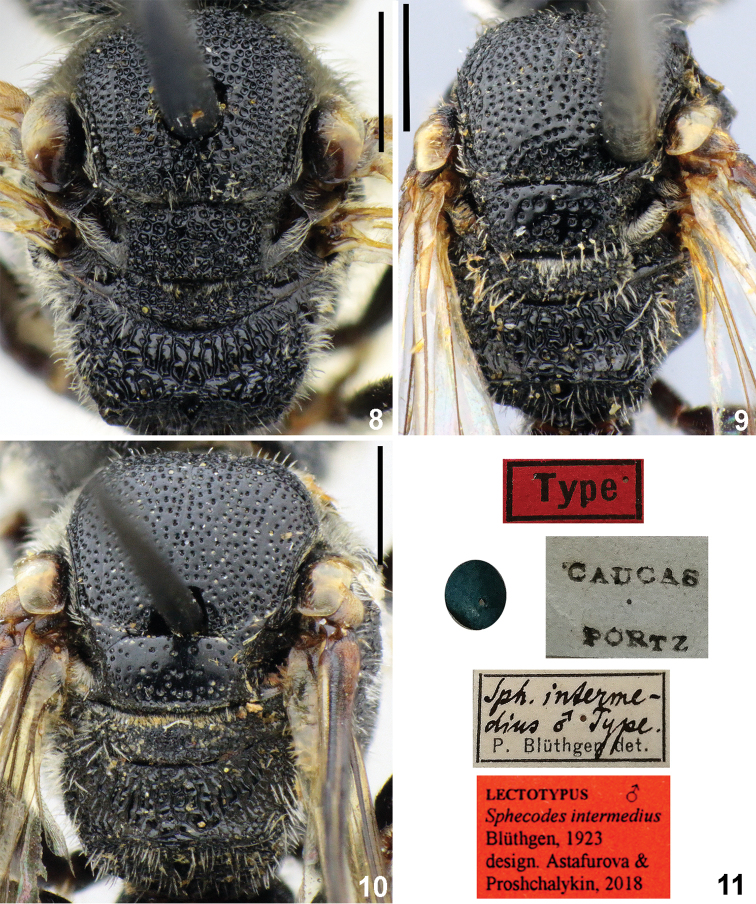
Mesosoma (**8–10**), dorsal view; lectotype labels (11). **8***Sphecodes scabricollis* Wesmael, male **9, 10***S. atlanticus* Warncke (**9** – male, **10** – female) **11***S. intermedius* Blüthgen, label of lectotype. Scale bars: 1.0 mm.

Metasoma (Fig. 19) with colouration varying from red on T1–T4 to entirely dark-brown; tergal discs with coarse and dense punctures (20–30 μm/ 0.5–2, sparser on anterior third of T1), marginal zone impunctate except on T1 with dense punctures (10–20 μm / 0.5–2); sterna finely tessellate with coarse setae pores; pygidial plate dull, as wide as metabasitarsus.

**Figures 12–15.**
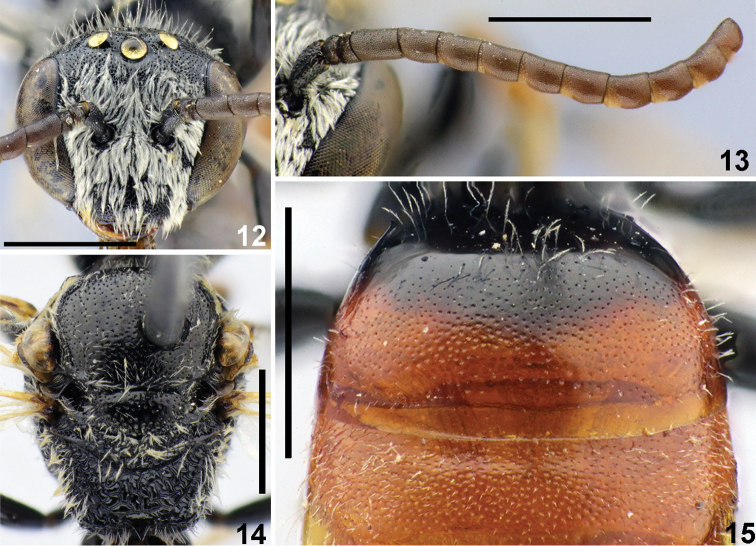
*Sphecodes dathei* Schwarz, male. **12** Head, frontal view **13** antenna, frontal view **14** mesosoma, dorsal view **15** T1, dorsal view. Scale bars: 1.0 mm.

**Figures 16–19.**
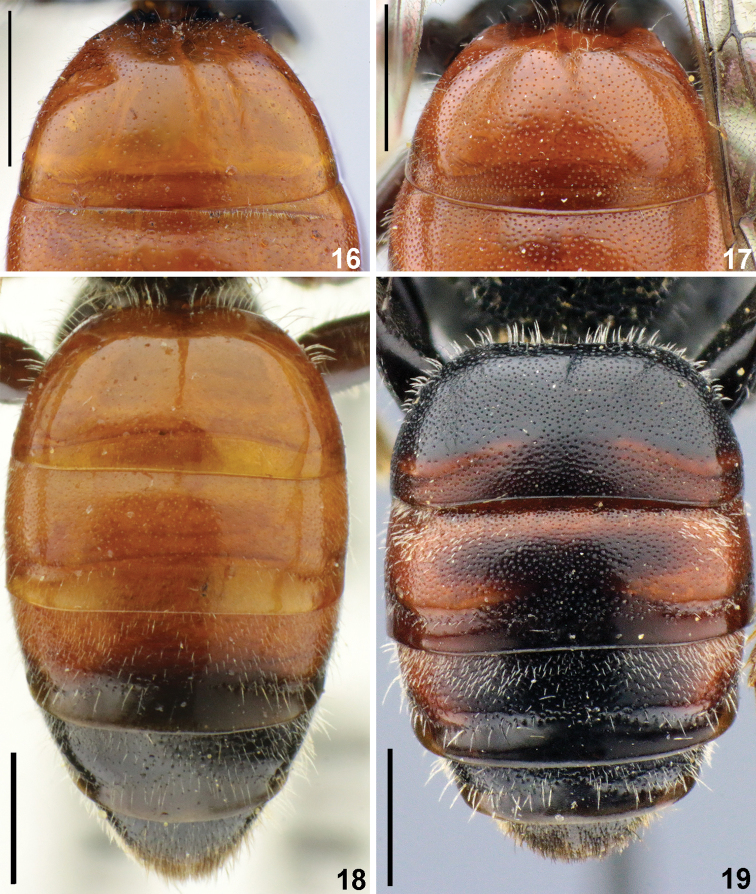
T1 (**16, 17**), metasoma (**18, 19**), females, dorsal view. **16***Sphecodes majalis* Pérez **17***S. barbatus* Blüthgen **18***S. scabricollis* Wesmael **19***S. atlanticus* Warncke. Scale bars: 1.0 mm.

##### Material examined.

SAUDI ARABIA: 6 ♂♂, Wadi Majarish (below Taif), 12.II.1983, K. Guichard (NHMUK 013380451, 013380453, 013380460, 013380462, 013380466, 013380459); 4 ♂♂, Fayfa, 200 m, 29.I. 1983, K. Guichard (NHMUK 013380452, 013380461, 013380463, 013380464); 1 ♂, Lodar, 800 m, 16.V.1967, K. Guichard (NHMUK 0133804446); 1 ♂, Abu Arish, 26.III.1980, K. Guichard (NHMUK 013380465); 2 ♀♀, 1 ♂, Abu Arish, Jizzan Hot Springs, 25.III.1980, K. Guichard (NHMUK 013380458, 013380441, 013380454), 1 ♂, idem, 28.I.1983 (NHMUK 013380450); 1 ♀, Wadi Maraba, 25.I.1983, 1000 m, K. Guichard (NHMUK 013380457); 1 ♀, Jeddah, Locust Research Station, 17.I.1972, A. Basha (NHMUK 013380442); YEMEN: 1 ♂, Usaifira, 1 mile N Ta’izz, 4.500 ft, 21.XII.1937, H. Scott, E. Britton (NHMUK 013380468), 1 ♂, Wadi Maytam, 12 km SE Ibb, 1600 m, 13°53'N, 44°18'E, 27.X.2005, J. Halada (OLBL/PCMS); 2 ♀♀, 3 ♂♂, Hawf NE Albhaydah, 200–730 m, 16°53'N, 53°05'E, 14.X.2005, J. Halada (OLBL/PCMS); 2 ♀♀, 4 ♂♂, 20 km S Taizz, 1200 m, 13°30'N, 43°57'E, 24.X.2005, J. Halada (OLBL/PCMS); 2 ♂♂, Jabal Bura, NEE Al Hudaydah, 200–800 m, 14°52'N, 43°24'E, 30.X-1.XI.2005, J. Halada (OLBL/PCMS); 1 ♂, Wadi Aniz, SSW Sana, 1520 m, 14°60'N, 44°09'E, 7.X.2005, J. Halada (OLBL/PCMS).

##### Distribution.

*Saudi Arabia, *Yemen; Algeria, the Canary Islands.

#### Sphecodes barbatus

Taxon classificationAnimaliaHymenopteraHalictidae

Blüthgen, 1923

517471C21052533F97CAE88F4771CD29

Figures 4 , 17

Sphecodes barbatus Blüthgen, 1923: 497–498, ♀ (holotype: ♀, Turkey, Ak-Chehir; ZSM).

##### Diagnosis.

*Sphecodes barbatus* is very similar to *S. majalis*. The two species are easily separable in the female, but males are difficult. The female *S. barbatus* differs from *S. majalis* by denser, distinctly plumose pubescence on paraocular areas and clypeus (Fig. 4) (sparser, weakly plumose or simple pubescence in *S. majalis*, Fig. 2) and by a distinctly (Fig. 17) punctate T1 (sparse and tiny punctures in *S. majalis*, Fig. 16).

##### Material examined.

SYRIA: 1 ♀, Syria, 40 km NE Damaskus, 22.V.1996, H. Halada (ZISP); 2 ♂♂, Slenfe, 1200 m, 19.IV.1986, K.M. Guichard, (NHMUK 013380371, 013380372).

##### Distribution.

*Syria; Greece, Turkey.

##### Remarks.

Warncke (1992) interpreted *Sphecodes barbatus* as a subspecies of *S. majalis* Pérez, 1903, but later this taxon was restored as a valid species (Bogusch and Straka 2014a).

#### Sphecodes dathei

Taxon classificationAnimaliaHymenopteraHalictidae

Schwarz, 2010

6D4AD2DBEB885FA39206B98C83009AB9

Figures 12–15 , 24

Sphecodes dathei Schwarz, 2010: 483–486, ♀, plates 1–12 (holotype: ♀, United Arab Emirates, Wadi Shawkah, 25°06'N, 56°02'E, 9–24.VI.2007, in water trap, A. van Harten leg.; SDEI), examined.

##### Diagnosis.

The species is similar to *Sphecodes crassus* Thomson, 1870 owing to the wide female metafemur (strongly enlarged in the basal half); strongly transverse female head; sparsely punctate mesoscutum in both sexes, weakly developed male antennal tyloids (usually covering less than 1/3 of ventral flagellar surfaces). The female of *Sphecodes dathei* differs from *S. crassus* by dense, apressed, snow-white, plumose pubescence obscuring integument in paraocular areas (sparse, simple pubescence not obscuring integument in *S. crassus*); the male differs by densely and relatively coarsely punctate T1 (in *S. crassus* T1 usually with a few fine punctures, rarely with relatively coarse and dense punctures). Both species have similar gonostylar shape, but *S. dathei* has a narrower, trapezoidal membranous portion of the gonostylus (wider, close to oval in *S. crassus*, Fig. 25).

**Figures 20, 21.**
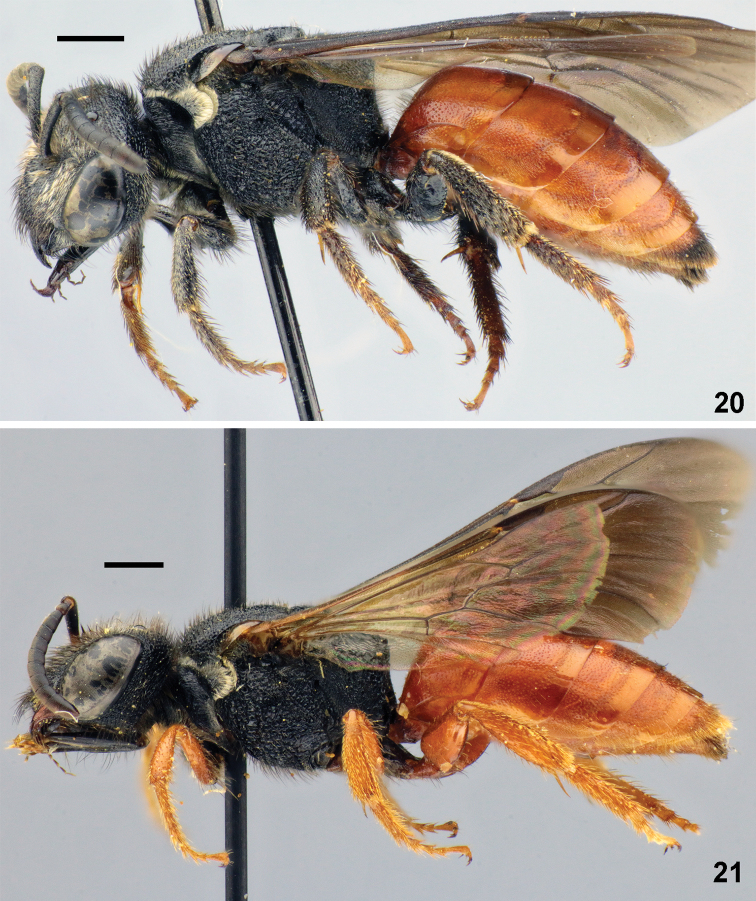
Habitus, females, lateral view. **20***Sphecodes albilabris* (Fabricius) **21***S. rubripes* Spinola. Scale bars: 1.0 mm.

**Figures 22–25.**
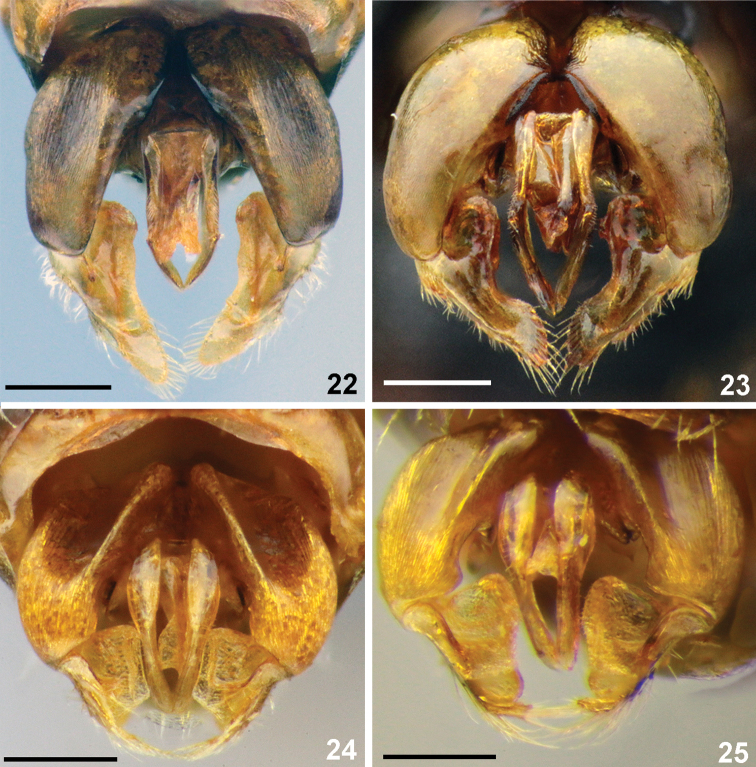
Genitalia, males, dorsal view. **22***Sphecodes atlanticus* Warncke **23***S. scabri collis* Wesmael **24***S. dathei* Schwarz **25***S. crassus* Thomson. Scale bars: 0.25 mm.

##### Description of hitherto unknown male.

Total body length 5.0–6.5 mm. Head (Fig. 12) black (except reddish mouthparts and brownish antenna); weakly transverse, 1.1 times as wide as long; vertex not elevated; distance from top of head to upper margin of lateral ocellus ca. two lateral ocellar diameters as seen in dorsal view; antenna (Fig. 13) reaches posterior margin of mesoscutum; F1 transverse, 0.6 times as long as wide; F2 long, 1.7 times as long as wide; remaining flagellomeres 1.2–1.3 times as long as wide; tyloids weakly developed (on F2–F4 covering less than 1/6 of ventral flagellar surfaces and from F5 onward covering less than 1/3); clypeus, frons, supraclypeal and paraocular areas with fine contiguous punctures (10–20 μm); ocello-ocular area and gena with shiny interspaces, punctures separated by 0.5–1 a puncture diameter; face below and above the antennal toruli with dense adpressed snow-white plumose pubescence obscuring integument; gena with similar pubescence, but not obscuring integument.

Mesosoma (Fig. 14) black; mesoscutum and mesoscutellum with punctures (20–25 μm) separated by 0.5–4 puncture diameters; mesepisternum and hypoepimeral area densely reticulate-rugose; propodeal triangle (Fig. 14) and vertical part of propodeum coarsely reticulate-rugose with shiny, smooth interspaces between wrinkles; lateral parts of propodeum coarse reticulate- to strigate-rugose with shiny interspaces between wrinkles; legs dark brown, but tarsi and partially tibia yellow or reddish. Hind wing costal margin with 5 hamuli.

Metasomal T1–T3 red (T1 black basally, T3 – apically); tergal discs (Fig. 15) with dense punctures (10–15 μm / 0.5–1), becoming sparse along marginal zone on T1; marginal zones smooth, impunctate; sterna with numerous microscopic setae pores; gonocoxite dorsally with a deep impression; membranous portion of gonostylus small, trapezoidal (Fig. 24).

##### Material examined.

SAUDI ARABIA: 1 ♀, Wadi Majarish, 800 m, 12.II.1983, K. Guichard (NHMUK 013380455); UNITED ARAB EMIRATES: 1 ♀, Hatta, 24.IV.1992 (NHMUK 013380414); 1 ♂, idem, 19–20.V.1988 (NHMUK 013380431); 7 ♂♂, idem, 14.IV.1990, I. Hammer (NHMUK 013380428, 013380429, 013380430, 013380432, 013380433, 013380434, 013380430); YEMEN: 1 ♀, Lawdar, NE Aden, 1140 m, 13°53'N, 45°48'E, 28.X.2005, J. Halada (OLBL/PCMS).

##### Published records.

Schwarz 2010: 483 (United Arab Emirates).

##### Distribution.

United Arab Emirates, *Saudi Arabia, *Yemen.

#### Sphecodes gibbus

Taxon classificationAnimaliaHymenopteraHalictidae

(Linnaeus, 1758)

5F913968CCE556978526570A1C4BB74D

Sphex gibba Linnaeus, 1758: 571, ♀ (syntypes: ♀♀, Sweden; ZMUK).
Apis glabra Füessly, 1775; Andrena ferruginea Olivier, 1789; Apis gibbosa Christ, 1791; Melitta sphecoides Kirby, 1802; M. picea Kirby, 1802; Andrena austriacaFabricius, 1804; Dichora analis Illiger, 1806; Sphecodes apicatus Smith, 1853; S. nigripennis Morawitz, 1876; S. sutor Nurse, 1903; S. gibbus var. rufispinosus Meyer, 1920; S. g. var. turkestanicus Meyer, 1920; S. castilianus Blüthgen, 1924; S. pergibbus Blüthgen, 1938; S. lustrans Cockerell, 1931; S. angarensis Cockerell, 1937 (Synonyms).

##### Diagnosis.

See Astafurova et al. 2018a: 17.

##### Material examined.

JORDAN: 1 ♀, Jordan Valey, Dayr Alla, 27.IV.1996, M. Halada (OLBL/PCMS); 1 ♀, N. Shuna env., 20–22.IV.1996; 1 ♀, idem, 29–30.IV.1996, M. Halada (OLBL/PCMS); SYRIA: 1 ♂, 20 km NE Latakia, 25.V.1996, M. Halada (OLBL/PCMS); ISRAEL: 1 ♀, Rehovot s.l., 29.IV.1975, K.M. Guichard (NHMUK 1975-248, 013380378); 1 ♀, Ein Gedi, 200 m, 11.III.1975, K.M. Guichard (NHMUK 1975-154, 013380379); 1 ♂, Jericho (Wadi Quilt), 250 m, 13–22.V.1975, K.M. Guichard (NHMUK 1975-248, 013380373); 1 ♀, Jericho, 200 m, 6–27.III.1975, K.M. Guichard (NHMUK 1975-154, 013380374).

##### Published records.

Warncke 1992: 30 (Israel).

##### Distribution.

Israel, *Jordan; North Africa, Europe (north to 63°), Russia (east to Yakutia), Turkey, Iran, Pakistan, Central Asia, Kazakhstan, Mongolia, NW China, India.

#### Sphecodes intermedius

Taxon classificationAnimaliaHymenopteraHalictidae

Blüthgen, 1923

7CC2FBA91AFE532A829B9A3365B2C600

Figure 11

Sphecodes intermedius Blüthgen, 1923: 500 (lectotype (**designated here**): ♂, Type <red label> // Caucas Portz // Sph. intermedius ♂, Type., P. Blüthgen det. // <blue circle> // Lectotypus, Sphecodes intermedius Blüthgen, 1923, ♂, des. Astafurova & Proshchalykin, 2018; paralectotype: ♀, Type <red label> Sph. intermedius ♀, Type, P. Blüthgen det. // Paralectotypus, Sphecodes intermedius Blüthgen, 1923, ♀, des. Astafurova & Proshchalykin, 2018; ISZP, examined, Fig. 11).
Sphecodes lactipennis Meyer, 1925 (Synonym).

##### Diagnosis.

See Astafurova et al. 2018a: 20.

##### Material examined.

UNITED ARAB EMIRATES: 1 ♂, Hatta (Hotel), 28. IV.1989, (NHMUK 013380370); 1 ♂, idem, 23.VIII.1991 (NHMUK 013380409); 1 ♂, idem, 14.IV.1990, I. L. Hamer [D. Baker det., 1992 as *S. punctatissimus* Meyer] (NHMUK 013380361); ISRAEL: 1 ♀, Jerusalim, 16.VII.1930, S. Bodenheimer [det. Blüthgen] (MNHB); 1 ♀, Tiberias, 200 m, 22.III.1975, K.M. Guichard (NHMUK 013380410); 1 ♀, Jericho (Hisham Palace), 200 m, 8.III.1975, K.M. Guichard (NHMUK 1975-248, 013380408); JORDAN: 1 ♀, N. Shuna env., 20–22.IV.1996; 1 ♀, idem, 29–30.IV.1996, M. Halada (OÖLM)

##### Published records.

Ascher and Pickering 2019 (Israel)

##### Distribution.

*United Arab Emirates, Israel, *Jordan; North Africa, South Europe (east to Ukraine), Russia (south of the European part, Urals), Caucasus, Turkey, Kazakhstan, Central Asia, Pakistan, China (Gansu).

##### Remarks.

*Sphecodes intermedius* Blüthgen, 1923 was described from specimens of both sexes collected in “Caucas” [Caucasus] (Fig. 11). There are two specimens (female and male) in ISZP from this locality, which correspond to the original description of P. Blüthgen. One of these specimens (male) is designated here as a lectotype of *S. intermedius* to avoid any confusion about the status of the type specimens and to properly diagnose this species.

#### Sphecodes longulus

Taxon classificationAnimaliaHymenopteraHalictidae

Hagens, 1882

43EBA5F314925AF7A2E67FBE8AB2EAED

Sphecodes longulus Hagens, 1882: 226, Fig. 25, ♂ (syntypes: ♂♂, Germany; ? Dominican monastery, Venlo, Nederland).
Sphecodes longulus var. eupidus Hagens, 1882; S. nitidulus Hagens, 1882; S. subfasciatus Blüthgen, 1934; S. amakusensis Yasumatsu & Hirashima, 1951; S. sabulosus Tsuneki, 1983; S. crassicornis Tsuneki, 1983; S. tsunekii Haneda, 1994 (Synonyms).

##### Diagnosis.

See Astafurova et al. 2018a: 21.

##### Material examined.

JORDAN: 1 ♀, 30 km N Tafila, 2.V.1996, M. Halada (OLBL/PCMS); 1 ♂, 20 km SW Madaba, 26.V.2007, 400 m, Z. Kejval (OLBL/PCMS); 1 ♂, Ajlun, 35 km W Jarash, 850 m, Z. Kejval (OLBL/PCMS); 1 ♀, 20 km S North Shuna Tall al Arbatin, 19.IV.1996, M. Halada (OLBL/PCMS); ISRAEL: 1 ♂, Dafna, 27.V.1991, K. Warncke (OLBL/PCMS); 1 ♂, North Galeleya, Nature Reserve ”Khule”, 23.V.1968, V. Trjapitzin (ZISP); 1 ♀, 5 km W Jericho, Wadi Qelet, St. Georg Mon., 6.V.1996, O. Niehuis (OLBL/PCMS); SYRIA: 1 ♂, Damask, 20–21.V.1980, M. Halada (OLBL/PCMS).

##### Published records.

Warncke 1992: 17 (Syria); Ascher and Pickering 2019 (Jordan).

##### Distribution.

*Israel, Jordan, Syria; Europe (north to Finland, Sweden, Denmark, England), Russia (east to Far East), Turkey, Iran, Central Asia, Kazakhstan, China, Japan.

#### Sphecodes majalis

Taxon classificationAnimaliaHymenopteraHalictidae

Pérez, 1903

8A5ED1678AF25BA98265B2151EB7EBBC

Figures 2 , 16

Sphecodes majalis Pérez, 1903: 219, ♀, ♂, (syntypes: ♀, ♂, France, Spain; MNHN).
Sphecodes gracilior Pérez, 1903; S. opacifrons Pérez, 1903; S. problematicus Schulz, 1906 (Synonyms).

##### Diagnosis.

Refer to the diagnosis *S. barbarus*, above.

##### Material examined.

JORDAN: 35 ♀ ♀, 5 ♂♂, 10 km N Petra, 3.V.1996, M. Halada (OLBL/PCMS); 2 ♀, 10 km N Jarash, 20.IV.2002, M. Snizek (OLBL/PCMS); 1 ♀, Ajlun S of Anjara, 27.IV.2002, M. Snizek (OLBL/PCMS).

##### Distribution.

*Jordan; North Africa, South Europe, Russia (south of the European part), Turkey, Iran.

#### Sphecodes marginatus

Taxon classificationAnimaliaHymenopteraHalictidae

Hagens, 1882

DCA18E56EF965AF78B75623ACCAAF279

Sphecodes marginatus Hagens, 1882: 223, Fig. 18, ♂ (syntypes: 2 ♂, Germany: Cleve; ? Dominican monastery, Venlo, Nederland).
Sphecodes atratus Hagens, 1882; S. nigritulus Hagens, 1882; S. biskrensis Pérez, 1903 (Synonyms).

##### Diagnosis.

This species belongs to the *miniatus* species group (*S. creticus* Warncke, 1992, *S. haladai* Warncke, 1992, *S. larochei* Warncke, 1992, *S. marginatus* Hagens, 1882, *S. miniatus* Hagens, 1882, *S. nomioidis* Pesenko, 1979, *S. schawrzi* Astafurova & Proshchalykin, 2014, and *S. sandykachis* Astafurova & Proshchalykin, 2018), with the same length and transverse F1–F3 in females. Among species of this group *S. marginatus* is most close to *S. miniatus* and *S. nomioidis* as they have a similar sculpture and structure of the body. Hence females of the three species are challenging to distinguish, but the male differs from the other two species by smaller triangular gonostylus. Differences between these three species are outlined by Bogusch and Straka (2012) and between females of this species group by Astafurova et al. (2018c).

##### Material examined.

ISRAEL: 1 ♀, 1 ♂, Jerusalim, 18.VI.1930; 1 ♂, idem, 10.VI.1931, S. Bodenheimer [det. Blüthgen] (MNHB); JORDAN: 1 ♀, W Jordan Valey, Mubalath, 27.IV.1996, M.Halada (OLBL/PCMS); 1 ♀, n. Shuna, 20–22.IV.1996, M. Halada(OLBL/PCMS); 2 ♂♂, NW of Ailun, 850 m, 20.V.2007, Z. Kejval (ZISP); 1 ♀, Jericho (Wadi Quilt), 250 m, 6.III.1975, K.M. Guichard (NHMUK 1975-154, 013380467).

##### Published records.

Schwarz 2010: 486 (United Arab Emirates); Ascher and Pickering 2019 (United Arab Emirates).

##### Distribution.

United Arab Emirates, *Israel, *Jordan; North Africa, Europe (north to Germany and Denmark, east to Belarus).

#### Sphecodes monilicornis

Taxon classificationAnimaliaHymenopteraHalictidae

(Kirby, 1802)

4AC06390842B516CAC0E5EECE61A4859

Melitta monilicornis Kirby, 1802: 47, ♂ (syntypes: ♂♂, England, NHMUK).
Sphecodes maculatus Lepeletier de Saint Fargeau, 1841; S. subquadratus Smith, 1845; S. ruficrus Dalla Torre, 1896; S. hanuman Nurse, 1903; S. monilicornis var. nigerrima Blüthgen, 1927; S. caucasicus Meyer, 1920; S. cephalotes Meyer, 1920; S. smyrnensis Meyer, 1920; S. monilicornis quadratus Meyer, 1920; S. monilicornis berberus Warncke, 1992; S. quadratus cephalotiformis Pittoni, 1950 (Synonyms).

##### Diagnosis.

See Astafurova et al. 2018a: 24.

##### Material examined.

SYRIA: 1 ♀, 50 km W Homs, 12.V.1996, M. Halada (OLBL/PCMS); 1 ♀, 60 km S Damask, Khabab, 14.V.1996, M. Halada (OLBL/PCMS); 1 ♀, 20 km S North Shuna Tall al Arbatin, 19.IV.1996, M. Halada (OLBL/PCMS); 1 ♀, 10 km W Jarasch, 1.V.1996, M. Halada (OLBL/PCMS); 2 ♀♀, Jisr ash Shunhur, 26.V.1996, M. Halada (OLBL/PCMS); 4 ♂♂, 20 km NE Latakia, 25.V.1996, M. Halada (OLBL/PCMS); 1 ♂, 30 km W Damask, 19.VI.2000, M. Halada (OLBL/PCMS); JORDAN: 1 ♀, Jarash env., 1.V.1996, M. Halada (OLBL/PCMS); 1 ♀, 10 km N Jarash, 1.V.1996, M. Halada (OLBL/PCMS); 1 ♂, 16 km WN Aijun, 600 m, 21.V.2077, Z. Kejval (OLBL/PCMS); 1 ♀, 10 km N Petra, 3.V.1996, M. Halada (OLBL/PCMS).

##### Published records.

Ascher and Pickering 2019 (Jordan).

##### Distribution.

Jordan, *Syria; North Africa, Europe (north to 64°), Russia (east to Far East), Caucasus, Turkey, Iran, Pakistan, Central Asia, Kazakhstan, Mongolia, China.

#### Sphecodes nomioidis

Taxon classificationAnimaliaHymenopteraHalictidae

Pesenko, 1979

5BE05D899B75554B805681C443A35A68

Sphecodes nomioidis Pesenko, 1979: 860, ♀ (holotype: ♀, Ukraine: Donetsk Province, Yenaktsevo, 10.VIII.1978, V. Radchenko leg.; ZISP).

##### Diagnosis.

Refer to diagnosis for *S. marginatus*, above.

##### Material examined.

UNITED ARAB EMIRATES: 4 ♂♂, Hatta, 14.IV.1990, I. Hamer (NHMUK 013380411, 013380415, 013380416, 013380417); OMAN: 1 ♂, Rostaq, 350 m, 21–31.III.1976, K. Guichard (NHMUK 013380443).

##### Published records.

Bogusch and Straka 2012: 14 (Jordan).

##### Distribution.

*United Arab Emirates, *Oman, Jordan; South and Central Europe (west to Austria), Ukraine, Russia (SW of the European part), Turkey.

#### Sphecodes olivieri

Taxon classificationAnimaliaHymenopteraHalictidae

Lepeletier de Saint Fargeau, 1825

8379E7A2BCD151EE998204A763B18992

Sphecodes olivieri Lepeletier de Saint Fargeau in Lepeletier de Saint Fargeau and Audinet-Serville, 1825: 448, ♂ (syntypes: ♂♂, ‘Arabie’).
Sphecodes collaris Spinola, 1843; S. hispanicus var. abyssinicus Sichel, 1865; S. ruficornis Sichel, 1865; S. punctulatus Sichel, 1865; S. subpunctulatus Sichel, 1865; S. rufithorax Morawitz, 1876; S. verticalis Hagens, 1882; S. desertus Nurse, 1903; S. chionospilus Cockerell, 1911; S. chionospilus var. sanguinatus Cockerell, 1911; S. tenuis Meyer, 1920; S. olivieri var. niveatus Meyer, 1925 (Synonyms).

##### Diagnosis.

See Astafurova et al. 2018a: 25.

##### Material examined.

UNITED ARAB EMIRATES, 1 ♂, Digdaga, 8.VIII.1984, J.N. Brown [D. Baker det, 92] (NHMUK 013380368); 1 ♂, Hatta (Hotel), 21.VIII.1987, I.L. Hamer [D. Baker det, 92] (NHMUK 013380366); 1 ♂, Soweihan Rd, 12.IV.1988, I.L. Hamer [D. Baker det, 92] (NHMUK 013380367); 1 ♂, Jebal Ali, 15.II.1991, I.L. Hamer [D. Baker det, 92] (NHMUK 013380361); SAUDI ARABIA, 1 ♀, 1♂, Jeddah, 15.II.1972, K.M. Guichard (NHMUK 013380399, 013380398); 1 ♀, 3 ♂♂, Riyadh area, 16–21.IV.1980, K.M. Guichard (NHMUK 013380456, 013380395, 013380396, 013380397); 1 ♂, Jeddah, 13.IV.1980 (NHMUK 013380393); 2 ♀♀, idem, 15.IV.1980, K.M. Guichard (NHMUK 013380392, 013380394); JORDAN: 1 ♀, 20 km W At Tafila, 1.VI.2007, Z. Kejval (OLBL/PCMS); OMAN, 1 ♀, Wadi Qurvat, Ag. Stn. 500 m, 5.III.1976, K. Guichard (NHMUK 013380383); 2 ♀♀, Tinaf, 650 m, 7.III.1976, K. Guichard (NHMUK 013380381, 013380380); 1 ♀, Rostaq, 350 m, 21–31.III.1976, K. Guichard (NHMUK 013380382);

ISRAEL, 1 ♂, Ein Bokek Zohar, 350 m, 25.V.1975, K.M. Guichard (NHMUK 1975-248, 013380385); ISRAEL: 1 ♀, Jericho (Wadi Kelt), 200 m, 6.III.1975, K.M. Guichard (NHMUK 1975-248, 013380387).

##### Published records.

Lepeletier de Saint Fargeau 1825: 448 (‘Arabie’); Warncke 1992: 46, map (Israel); Dathe 2009: 385 (United Arab Emirates); Schwarz 2010: 486 (United Arab Emirates); Ascher and Pickering 2019 (United Arab Emirates, Qatar).

##### Distribution.

United Arab Emirates, *Oman, Qatar, *Saudi Arabia, Israel, *Jordan; North Africa, South Europe, Russia (South of European part), Turkey, Caucasus, Iran, Pakistan, Central Asia, Kazakhstan, NW China.

#### Sphecodes pellucidus

Taxon classificationAnimaliaHymenopteraHalictidae

Smith, 1845

80F345845F915F009BB3FD1A6E8B1BBD

Sphecodes pellucidus Smith, 1845: 1014, ♀, ♂ (syntypes: ♀♀, ♂♂, England; NHMUK).
Sphecodes pilifrons Thomson, 1870; S. brevicornis Hagens, 1874; S. volatilis Smith, 1879; S. pellucidus var. algirus Alfken, 1914; S. pellucidus var. hybridus Blüthgen, 1924; S. pellucidus var. niveipennis Meyer, 1925 (Synonyms).

##### Diagnosis.

See Astafurova et al. 2018a: 27.

##### Material examined.

SYRIA: 1 ♀, 30 km N Dara, Nawa, 18.V.1996, M. Halada (OLBL/PCMS); JORDAN: 1 ♀, Jordan valley, S. Shuna, 17.IV.1996, M. Halada (OLBL/PCMS); 1 ♀, 10 km N Petra, 3.V.1996, M. Halada (OLBL/PCMS).

##### Distribution.

*Jordan, *Syria; North Africa, Europe (north to 66°), Russia (east to Far East), Turkey, Iran, Central Asia, Kazakhstan, Mongolia, China.

#### Sphecodes pinguiculus

Taxon classificationAnimaliaHymenopteraHalictidae

Pérez, 1903

1BA02AED9E255C609B3F70042BC8AE7F

Sphecodes pinguiculus Pérez, 1903: CCXX, ♀ (syntypes: ♀♀, Spain: Catalonia; MNHN).
Sphecodes sareptensis Meyer, 1922; S. excellens Meyer, 1922; S. punctatissimus Meyer, 1922; S. hungaricus Blüthgen, 1923; S. coelebs Blüthgen, 1923; S. consobrinus Blüthgen, 1923; S. persicus Blüthgen, 1924; S. capverdensis Pauly & La Roche, 2002 (Synonyms).

##### Diagnosis.

See Astafurova et al. 2018a: 30.

##### Material examined.

SYRIA: 1 ♂, 80 km E Palmira, 450 m, 22.IV.1992, K. Warncke (OÖLM); SAUDI ARABIA, 1 ♀, Hofut, 145 m, 21–6.IV.1980, K. Guichard (NHMUK 013380359); 1 ♀, Hatta, 10.IV.1983, I.L. Hamer (NHMUK 013380389); 1 ♀, idem, 6.VI.1986, I.L. Hamer (NHMUK 013380390); 2 ♀♀, Khor-Fakkan, 20.III.1987, I.L. Hamer (NHMUK 013380364, 013380377); 1 ♀, Soweihan Rd, 12.IV.1988, I.L. Hamer (NHMUK 013380391); OMAN, 1 ♀, Wadi Qurvat, Ag. Stn. 500 m, 5.III.1976, K. Guichard (NHMUK 013380407); 2 ♀♀, Rostaq, 350 m, 21–31.III.1976, K. Guichard (NHMUK 013380405, 013380406);

ISRAEL: 1 ♂, Tel-Aviv, 22.IV.1966, Bytinski-Salz (OLBL/PCMS); 3 ♀♀, Jericho (Hisham Palace), 200 m, 8.III.1975, K.M. Guichard (NHMUK 1975-248, 013380408).

##### Published records.

Schwarz 2010: 486 (United Arab Emirates, Israel); Ascher and Pickering 2019 (Israel, United Arab Emirates).

##### Distribution.

United Arab Emirates, *Oman, *Saudi Arabia, Israel, *Syria; Cape Verde Islands, North Africa, South Europe, Russia (east to Buryatia), Turkey, Iran, Central Asia, Kazakhstan, Mongolia, North China.

#### Sphecodes puncticeps

Taxon classificationAnimaliaHymenopteraHalictidae

Thomson, 1870

E61D565668005FD398FBE21910268C45

Sphecodes puncticeps Thomson, 1870: 99, ♀, ♂ (syntypes: ♀♀, ♂♂, Sweden; MZLU).
Sphecodes bituberculatus Pérez, 1903; S. opacifrons Pérez, 1903; S. puncticeps var. cretanus Strand, 1921 (Synonyms).

##### Diagnosis.

See Astafurova et al. 2018a: 31.

##### Material examined.

SAUDI ARABIA: 1 ♂, Riyadh area, 16–21.IV.1980, K.M. Guichard (NHMUK 013380403); JORDAN: 1 ♀, Jordan valley, S. Shuna, 17.IV.1996, M. Halada (OLBL/PCMS); 2 ♀, 3 ♂♂, Jordan valley, Dayr Alla, 27.IV.1996, M. Halada (OLBL/PCMS); 1 ♀, 10 km N Petra, 3.V.1996, M. Halada (OLBL/PCMS); 1 ♀, 10 km SE Suwayda Kafr, 19.V.1996, M. Halada (OLBL/PCMS); SYRIA: 1 ♀, Latakia s.l., 17.VI.1986, K. Guichard (NHMUK 013380360); 2 ♀♀, 10 km SE Suwayda Kafr, 19.V.1996, M. Halada (OLBL/PCMS); ISRAEL: 2 ♂♂, Jerusalem, 21.IX.1922, P.A. Buxton (NHMUK 013380401, 013380400); 1 ♂, Rehovot s.l., 29.IV.1975, K.M. Guichard (NHMUK 1975-248, 013380404); 3 ♂♂, Jericho (Wadi Quilt), 250 m, 13–22.V.1975, K.M. Guichard (NHMUK 1975-248, 013380365, 013380376, 013380402).

##### Published records.

Warncke 1992: 19 (Israel); Ascher and Pickering 2019 (Israel).

##### Distribution.

*Saudi Arabia, Israel, *Jordan, *Syria; North Africa, Europe (north to Finland and Sweden), Russia (east to Far East), Turkey, Iran, Central Asia, Kazakhstan, Mongolia.

#### Sphecodes rubicundus

Taxon classificationAnimaliaHymenopteraHalictidae

Hagens, 1875

68AD66DC2D4A583CBEFB7835049C7E1E

Sphecodes rubicundus Hagens, 1875: 318 (syntypes: ♂♂, ♀♀, Germany; ? Dominican monastery, Venlo, Nederland).
Sphecodes rubicundus altisilesiacus Torka, 1927 (Synonyms).

##### Diagnosis.

The female of this species as well as *S. ruficrus* is most close to *S. pellucidus* and *S. ephippius* owing to a densely punctate head and mesosoma, relative wide pygidial plate and impunctate T1, but differs by having a distinctly elevated vertex with the distance between vertex and upper margin of lateral ocellus at least a lateral ocellar diameter as seen in frontal view (versus 0.2–0.5). *S. rubicundus* differs from *S. ruficrus* by white pubescence of head and mesosoma (with brown setae in *S. ruficrus*) and a less curved basal (M) vein in hind wing. The male most closely resembles *S. pesenkoi* Astafurova & Proshchalykin, 2018 and *S. ruficrus* (Erichson, 1835) owing to a similar gonostylar shape (elongate, spoon-shaped). The male of *S. rubicundus* differs from *S. pesenkoi* by an areolate mesoscutum (versus punctures separated by 1–3 puncture diameters) and coarsely and densely punctate T1 (a few fine punctures in *S. pesenkoi*).

According to the phylogenetic analysis (Habermannová et al. 2013) *Sphecodes rubicundus*, *S. ruficrus*, *S. pellucidus*, and *S. ephippius* belong to the same clade. Relationship between these species also is well supported by morphological characters.

##### Material examined.

ISRAEL, 2 ♂♂, Jerusalem, 800 m, 20.III.1975, K.M. Guichard (NHMUK 1975-154, 013380388, 013380386); 1 ♀, Tiberias, 200 m, 22.III.1975, K.M. Guichard (NHMUK 1975-154, 013380384); 1 ♀, Jerusalem, 20.III.1993, D. Ahal (OLBL/PCMS).

##### Distribution.

*Israel; Europe (north to 56°), Russia (south of the European part), Turkey, Caucasus, Iran.

#### Sphecodes rubripes

Taxon classificationAnimaliaHymenopteraHalictidae

Spinola, 1839

AED78E1E647A59999CE12B44195BAF69

Figures 6 , 21

Sphecodes rubripes Spinola, 1839: 512, ♀ (syntypes: ♀♀, Cyprus; MRSN).
Sphecodes africanus Lepeletier, 1841; S. rufipennis Cockerell, 1931, S. atrescens Cockerell, 1931 (Synonyms).

##### Diagnosis.

The female of *S. rubripes* differs from *S. albilabris* by the pubescence of paraocular area (Fig. 6) with brown erect setae not obscuring integument (versus white plumose appressed pubescence obscuring integument usually with admixture of brownish erect setae in *S. albilabris*, Fig. 7). Both sexes also differ by mainly red legs, except brown coxae and trochanters, Fig. 21 (at most reddish tarsi and tibia in *S. albilabris*, Fig. 20). These two species also differ in phenology (males of *S. rubripes* were recorded in the early spring while males of *S. albilabris* were found in the summer) and have different hosts (Bogusch and Straka 2012, Cross 2017). *S. albilabris* is widespread in the Palaearctic from the Atlantic Ocean to Russian Far East; however, the distribution of the species in the Mediterranean Region is unclear due to confusion with *S. rubripes.* The past records of *S. albilabris* from Israel and Syria refer to *S. rubripes.* We examined material of *S. albilabris* from Morocco and Tunisia, but we do not have any specimens of *S. albilabris* from the Arabian Peninsula or surrounding lands.

##### Material examined.

JORDAN: 1 ♀, 10 km N Petra, 3.V.1996, M. Halada (OLBL/PCMS).

##### Published records.

Meyer 1924: 3 (Syria, as *S. fuscipennis rubripes*); Warncke 1992: 31 (Israel, as *S. albilabris rubripes*); Ascher and Pickering 2019 (Israel, as *S. albilabris* (Fabricius)).

##### Distribution.

*Jordan, Israel, Syria; North Africa, South-Western Europe, Cyprus.

##### Remarks.

Mayer (1924) and later Warncke (1992) interpreted *Sphecodes rubripes* as a subspecies of *S. albilabris* (Fabricius, 1793), but this taxon was restored as a valid species (Bogusch and Straka 2014b).

#### Sphecodes ruficrus

Taxon classificationAnimaliaHymenopteraHalictidae

(Erichson, 1835)

4DD92252598B52318ED7B28E7E56E63D

Dichroa ruficrus Erichson, 1835: 101, ♀, (syntypes: ♀♀, Spain; ZMHB).
Sphecodes hispanicus Wesmael, 1836; S. rufipes Smith, 1853; S. gibbus var. tunetanus Gribodo, 1894; S. atrohirtus Pérez, 1903 (Synonyms).

##### Diagnosis.

Refer to diagnosis for *S. rubicundus*, above.

##### Material examined.

JORDAN: 1 ♀, W Jordan Valley, env. of S. Shuna, 17.IV.1996, M. Halada (OLBL/PCMS).

##### Published records.

Warncke 1992: 21 (Israel); Ascher and Pickering 2019 (Israel).

##### Distribution.

Israel, *Jordan; North Africa, southwestern Europe.

##### Remarks.

Russia is mistakenly listed as within the distribution by Bogusch and Straka (2012) as well as Turkey and Armenia by Özbek et al. (2015) due to confusion with *S. ruficrus rubicundus* sensu Warncke (1992).

#### Sphecodes rufiventris

Taxon classificationAnimaliaHymenopteraHalictidae

(Panzer, 1798)

EA4EA9F64BA55BF3A1FC3AF064E687E7

Tiphia rufiventris Panzer, 1798: 4, ♀ (syntypes: ♀♀, Germany; ZMHB).
Sphecodes subovalis Schenck, 1853; S. brevis Hagens, 1875; S. singularis Meyer, 1920; S. combinatus Blüthgen, 1927; S. subovalis austrinus Erlandsson, 1979; S. rufiventris hethiticus Warncke, 1992 (Synonyms).

##### Diagnosis.

See Astafurova et al. 2018a: 34.

##### Material examined.

JORDAN: 1 ♂, W Jordan Valey, Mubalath, 27.IV.1996, M. Halada (OLBL/PCMS).

##### Published records.

Ascher and Pickering 2019 (Israel).

##### Distribution.

Israel, *Jordan; North Africa, Europe, (north to 57°), Russia (east to Khakassia Republic), Turkey, Iran, Central Asia, Kazakhstan.

#### Sphecodes schenckii

Taxon classificationAnimaliaHymenopteraHalictidae

Hagens, 1882

30EA22E5954652D3BF5B8627DB0398EC

Sphecodes schenckii Hagens, 1882: 217, ♂ (holotype: ♂, no locality, Rudow leg. [see Blüthgen 1923: 444]; MNHB).
Sphecodes sulcicollis Pérez, 1903; S. caspicus Meyer, 1920 (Synonyms).

##### Diagnosis.

See Astafurova and Proshchalykin 2017b: 274.

##### Material examined.

JORDAN: 1 ♂, NW Ajlun, 850 m, 20.V.2007, Z. Kejval (OLBL/PCMS) ; 1 ♀, 10 km N Petra, 3.V.1996, M. Halada (OLBL/PCMS); SYRIA: 3 ♂♂, 20 km NE Latakia, 25.V.1996, M. Halada (OLBL/PCMS).

##### Published records.

Warncke 1992: 27 (Israel, as *Sphecodes schenckii caspicus* Meyer); Ascher and Pickering 2019 (Israel).

##### Distribution.

Israel, *Jordan, *Syria; Europe (north to Germany), Russia (European part), Turkey, Caucasus, ? Iran.

#### Sphecodes tadschicus

Taxon classificationAnimaliaHymenopteraHalictidae

Blüthgen, 1935

C6671BF631855F19A9D51ED3E1E89547

Sphecodes tadschicus Blüthgen in Popov, 1935: 366, ♂, ♀ (holotype: ♂, near Kulab [Tajikistan], 25.VII.1935, V. Popov leg.; ZISP).

##### Diagnosis.

See Astafurova et al. 2018a: 39.

##### Material examined.

ISRAEL: 1 ♀, 8 ♂♂, Jerusalem, 10–25.VIII.1960, Bytinski (MNHB).

##### Distribution.

*Israel; Turkey, Iran, Central Asia, Kazakhstan.

#### Sphecodes turanicus

Taxon classificationAnimaliaHymenopteraHalictidae

Astafurova & Proshchalykin, 2017

7D2BE7510D0857F28E38D5D19D9C4E52

Sphecodes turanicus Astafurova & Proshchalykin, 2017b: 274, ♂, ♀ (holotype: ♀, Turkmenistan, Chardzhou, 16.IV.1988, Dialentov leg.; ZISP).

##### Diagnosis.

See Astafurova et al. 2018a: 41.

##### Material examined.

SAUDI ARABIA: 1 ♂, Riyadh, El Ha’ir, 16–21.IV.1980, K.M. Guichard (NHMUK 013380449).

##### Distribution.

*Saudi Arabia; Central Asia, Kazakhstan, China (Gansu).

#### Sphecodes villosulus

Taxon classificationAnimaliaHymenopteraHalictidae

Schwarz, 2010

D12FCFAA24895241B1E7149122E79AA7

Sphecodes villosulus Schwarz, 2010: 486–491, ♀, ♂ (holotype: ♀, United Arab Emirates, Dubai, Nakhalai, 28–30.IV.1984, in Malaise trap, E. Sugden leg.; BLCU).

##### Diagnosis.

This species differs from other small Palaearctic species with 5–6 hamuli in the hind wing by having a unique combination of simple mandibles and the male gonocoxite dorsally with an impression. The female is closest to *S. armeniacus* owing to dense appressed snow-white pubescence obscuring the integument on face, a transverse head and sparsely punctate mesoscutum, but differs from this species by sparser and finer punctate ocello-ocular area (3–5 μm / 2–3 versus 5–10 μm / 1–2) and strongly transverse F3 (almost square in *S. armeniacus*). The male of *S. villosulus* recalls *S. miniatus* in the rectangular gonostylar shape, but clearly differs from this species by the less developed tyloids on the flagellomeres extending to approximately a half of ventral flagellar surfaces (versus those across 4/5).

##### Material examined.

UNITED ARAB EMIRATES: 1 ♀, 1♂, Abu Dhabi, 30.I.1987, I. Hamer (NHMUK 013380419, 013380423); 1 ♀, 2♂♂, Abu Dhabi, 31.III.1987 (NHMUK 013380418, 013380421, 013380420); 1 ♀, idem, 10.IV.1987, I. Hamer (NHMUK 013380422); 1 ♀, Hatta, 20.XII.1990 (NHMUK 013380424); 2 ♂♂, idem, 23.VIII.1991 (NHMUK 013380426, 013380427); 1 ♀, idem, 5.III.1993, I. Hamer (NHMUK 013380425); 3 ♀♀, North Ras, Al Khaimah, 17.II.2018 (M. Mokrousov) (ZISP); SAUDI ARABIA: 1 ♀, Riyadh, El Ha’ir, 19.III.1980, K.M. Guichard (NHMUK 013380448); OMAN: 1 ♀, Rostaq, 350 m, 21–31.III.1976, K.M.Guichard (NHMUK 013380444).

##### Published records.

Schwarz 2010: 486 (United Arab Emirates); Ascher and Pickering 2019 (United Arab Emirates).

##### Distribution.

United Arab Emirates, *Oman, *Saudi Arabia.

## Discussion

In total, 26 species of *Sphecodes* are recorded from the Arabian Peninsula and surrounding lands (Israel, Jordan and Syria) (Table 1). This is a comparable number to the Iranian fauna, but distinctly less in comparison with the adjacent fauna of Turkey, North Africa and Central Asia (Table 3).

**Table 3.** List of *Sphecodes* species recorded in Arabian Peninsula and surrounding lands (AP+SL), Turkey, Iran, North Africa (Morocco, Algeria, Libya, Tunisia, Egypt) and Central Asia (Kazakhstan, Kyrgyzstan, Uzbekistan, Turkmenistan, Tajikistan).

	**Sphecodes species**	**AP+SL**	**Turkey**	**Iran**	**North Africa**	**Central Asia**
1	*S. albilabris* (Fabricius, 1793)	–	+	+	+	+
2	*S. alternatus* Smith, 1853	+	+	+	+	+
3	*S. anatolicus* Warncke, 1992	–	+	+	–	+
4	*S. armeniacus* Warncke, 1992	–	+	–	+	+
5	*S. atlanticus* Warncke, 1992	+	–	–	+	–
6	*S. atlassa* Warncke, 1992	–	–	–	+	–
7	*S. barbatus* Blüthgen, 1923	+	+	–	–	–
8	*S. crassanus* Warncke, 1992	–	+	–	–	+
9	*S. crassus* Thomson, 1870	–	+	+	+	+
10	*S. cristatus* Hagens, 1882	–	+	–	–	+
11	*S. croaticus* Meyer, 1922	–	+	+	+	+
12	*S. dathei* Schwarz, 2010	+	–	–	–	–
13	*S. dusmeti* Blüthgen, 1924	+	+	–	+	–
14	*S. ebmeri* Astafurova & Proshchalykin, 2018	–	–	+	–	–
15	*S. ephippius* (Linné, 1767)	+	+	+	–	+
16	*S. ferruginatus* Hagens, 1882	–	+	–	–	+
17	*S. geoffrellus* (Kirby, 1802)	–	+	–	+	+
19	*S. gibbus* (Linnaeus, 1758)	+	+	+	+	+
20	*S. hakkariensis* Warncke, 1992	–	+	–	–	+
21	*S. haladai* Warncke, 1992	–	–	+	+	+
22	*S. hyalinatus* Hagens, 1882	–	–	–	–	+
23	*S. hirtellus* Blüthgen, 1923	–	–	–	+	–
24	*S. intermedius* Blüthgen, 1923	+	+	–	+	+
25	*S. longulus* Hagens, 1882	+	+	+	–	+
27	*S. longuloides* Blüthgen, 1923	+	–	–	+	–
28	*S. majalis* Pérez, 1903	+	+	+	+	–
29	*S. marginatus* Hagens, 1882	+	–	–	+	–
30	*S. monilicornis* (Kirby, 1802)	+	+	+	+	+
31	*S. niger* Hagens, 1874	–	+	–	–	–
32	*S. nomioidis* Pesenko, 1979	+	+	–	–	–
33	*S. nurekensis* Warncke, 1992	–	–	–	–	+
34	*S. olivieri* Lepeletier de Saint Fargeau, 1825	+	+	+	+	+
35	*S. pectoralis* Morawitz, 1876	–	–	+	–	+
36	*S. pellucidus* Smith, 1845	+	+	+	+	+
37	*S. pesenkoi* Astafurova & Proshchalykin, 2018	–	–	–	–	+
38	*S. pinguiculus* Pérez, 1903	+	+	+	+	+
39	*S. pseudofasciatus* Blüthgen, 1925	–	+	–	+	–
40	*S. puncticeps* Thomson, 1870	+	+	+	+	+
41	*S. reticulatus* Thomson, 1870	–	+	+	–	+
42	*S. rubicundus* Hagens, 1875	+	+	+	–	–
43	*S. rubripes* Spinola, 1839	+	–	–	+	–
44	*S. ruficrus* (Erichson, 1835)	+	–	–	+	–
45	*S. rufiventris* (Panzer, 1798)	+	+	+	+	+
46	*S. sandykachis* Astafurova & Proshchalykin, 2018	–	–	–	–	+
47	*S. saxicolus* Warncke, 1992	–	–	+	–	+
48	*S. scabricollis* Wesmael, 1835	–	+	+	–	+
49	*S. schenckii* Hagens, 1882	+	+	+	–	–
50	*S. schwarzi* Astafurova & Proshchalykin, 2015	–	–	–	–	+
51	*S. spinulosus* Hagens, 1875	–	+	+	+	+
52	*S. tadschicus* Blüthgen, 1935	+	+	+	–	+
53	*S. trjapitzini* Astafurova & Proshchalykin, 2018	–	–	–	–	+
54	*S. turanicus* Astafurova & Proshchalykin, 2017	+	–	–	–	+
55	*S. zangherii* Noskiewicz, 1931	–	+	–	–	–
56	*S. villosulus* Schwarz, 2010	+	–	–	–	–
**Total**:		**26**	**34**	**25**	**26**	**35**

The distribution of Sphecodes species are given according to Özbek et al. 2015 (Turkey), Astafurova et al. 2018d (Iran), Warncke 1992 (North Africa), Astafurova and Proshchalykin 2017b and Astafurova et al. 2018a, c (Central Asia).

The *Sphecodes* fauna of the Arabian Peninsula and surrounding lands is a complex of Mediterranean, Sahara-Gobian, endemic, and species widespread in the Palaearctic region. Eight species, namely *S. alternatus*, *S. ephippius*, *S. gibbus*, *S. longulus*, *S. monilicornis*, *S. marginatus*, *S. pellucidus*, and *S. puncticeps* are widespread from north to south of the Palaearctic region and occur in biomes ranging from forest to desert. However, two of these (*S. marginatus* and *S. puncticeps*) are recorded from the Arabian Peninsula and the remainder all are found only in Mediterranean areas.

*Sphecodes majalis*, *S. schenckii* Hagens, *S. rubicundus*, and *S. nomioidis* are steppe species, distributed in Europe, Turkey and the Caucasus to Iran. Of them, only *S. nomioidis* is recorded from the Arabian Peninsula.

*Sphecodes olivieri*, *S. intermedius*, *S. rufiventris*, and *S. pinguiculus* are widespread from steppe to desert in the Western Palaearctic. Of these only *S. rufiventris* is not recorded from the Arabian Peninsula.

*Sphecodes barbatus*, *S. rubripes*, and *S. ruficrus* are possibly purely Mediterranean species not reaching the Arabian Peninsula. In contrast, *S. atlanticus* turns out to be Sahara-Arabian. *Sphecodes dusmeti* and *S. longuloides* are Mediterranean-Arabian species.

*Sphecodes tadschicus* and *S. turanicus* are Irano-Turanian species reaching the Arabian Peninsula.

Finally, two species, *S. dathei* and *S. villosulus* are endemic to the Arabian Peninsula.

Although the Arabian fauna of the genus is not fully studied it is now clear that the Arabian fauna differs from that of the Mediterranean; of 26 recorded species only six (*S. olivieri*, *S. intermedius*, *S. marginatus*, *S. nomioidis*, *S. pinguiculus*, and *S. puncticeps*) are common to both and these are all widespread in the Western Palaearctic.

## Supplementary Material

XML Treatment for Sphecodes alternatus

XML Treatment for Sphecodes atlanticus

XML Treatment for Sphecodes barbatus

XML Treatment for Sphecodes dathei

XML Treatment for Sphecodes gibbus

XML Treatment for Sphecodes intermedius

XML Treatment for Sphecodes longulus

XML Treatment for Sphecodes majalis

XML Treatment for Sphecodes marginatus

XML Treatment for Sphecodes monilicornis

XML Treatment for Sphecodes nomioidis

XML Treatment for Sphecodes olivieri

XML Treatment for Sphecodes pellucidus

XML Treatment for Sphecodes pinguiculus

XML Treatment for Sphecodes puncticeps

XML Treatment for Sphecodes rubicundus

XML Treatment for Sphecodes rubripes

XML Treatment for Sphecodes ruficrus

XML Treatment for Sphecodes rufiventris

XML Treatment for Sphecodes schenckii

XML Treatment for Sphecodes tadschicus

XML Treatment for Sphecodes turanicus

XML Treatment for Sphecodes villosulus
